# Diseases Admitted under the Care of the Physicians of the Westminster Hospital, from the 20th of November to the 18th of December 1799

**Published:** 1800-01

**Authors:** 


*Diſeaſes admitted under the care of the Phyſicians of the Weſtminſter Hoſpital, from the 20th of November to the* 18*th of December* 1799.

| | | | |
|---|---|---|---|
| Fevers | 10 | Hæmoptoe | 2 |
| Pleuriſy | 1 | Hæmorrhois | 3 |
| Scarlatina | 1 | Hypochondriaſis | 1 |
| Intermittent | 1 | Hyſteria | 2 |
| Amenorrhœa | 3 | Impetigo | 2 |
| Anaſarca | 4 | Itch | 2 |
| Aſthma | 2 | Jaundice | 1 |
| Catarrh | 5 | Lepra Græcorum | 1 |
| Cephalæa | 2 | Lumbago | 1 |
| Cholera | 1 | Phthiſis | 4 |
| Colic | 1 | Paralyſis | 3 |
| Cough | 12 | Quinzey | 2 |
| Diarrhœa | 3 | Rheumatiſm | 7 |
| Dyſpepſia | 4 | Struma | 1 |
| Enterodynia | 3 | Tinea | 1 |
| Erythenia | 1 | Worms | 1 |
| Gaſtrodynia | 1 | | |

Though febrile affections have become more frequent during the laſt month, they have not aſſumed any appearance of malignancy in this part of the metropolis.

